# Supplementary material for: Nucleotide– and Mal3-dependent changes in fission yeast microtubules suggest a structural plasticity view of dynamics
Source: Nat Commun. 2017 Dec 13;8:2110. doi: 10.1038/s41467-017-02241-5 (PMC5727398; doi:10.1038/s41467-017-02241-5)
Supplement: Supplementary file 1 — Supplementary Information [file 41467_2017_2241_MOESM1_ESM.pdf]

# SUPPLEMENTARY INFORMATION

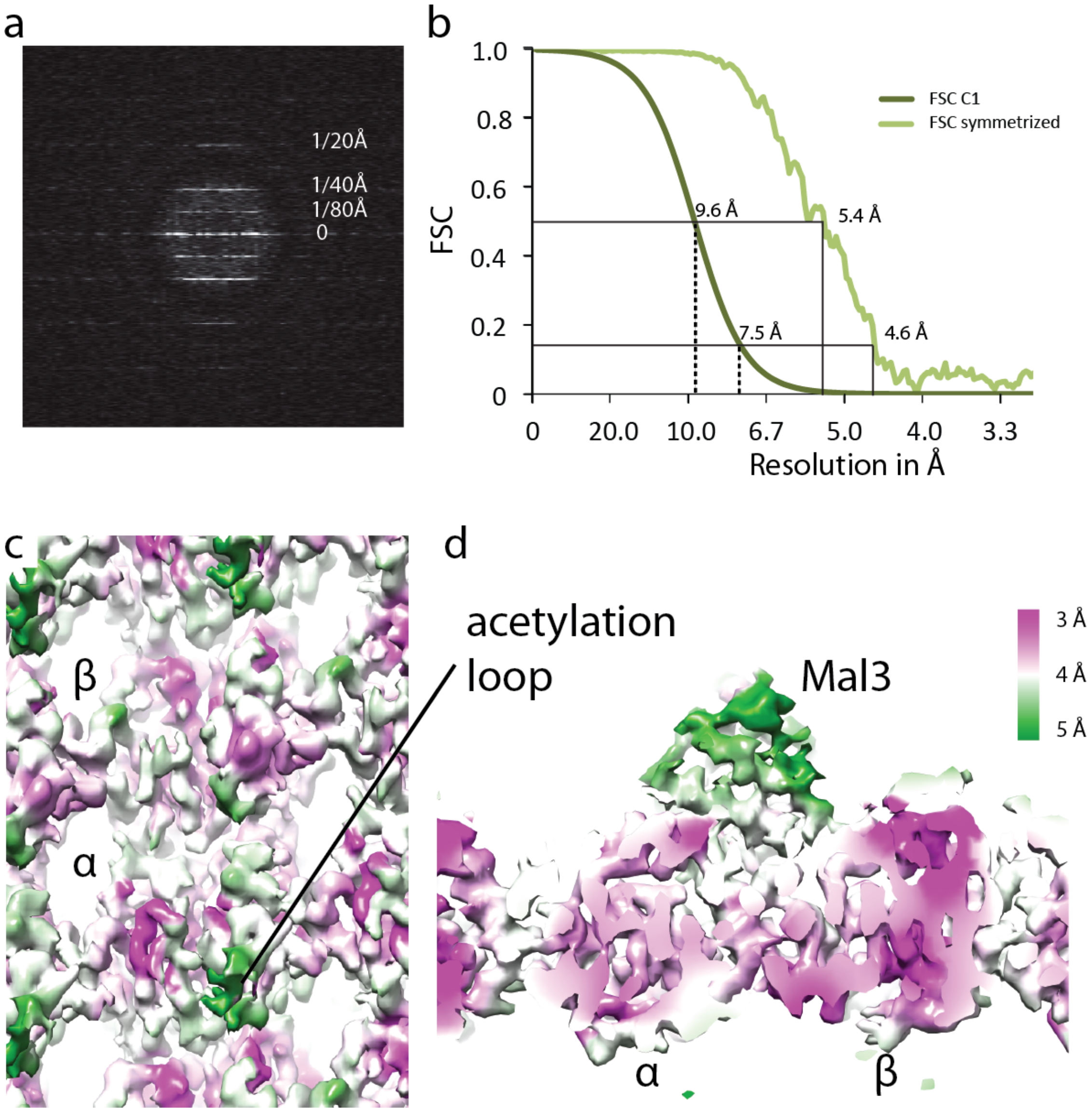


**Supplementary Figure 1. Evaluation of the resolutions of the Mal3-bound Sp_MT reconstructions. a**. Exemplar Fourier transform of the sum of all individually boxed particles from one Mal3-bound Sp_MT used for reconstruction, zoomed in to show a strong layer line at 1/80 $Å^{-1}$ due to Mal3 binding to every tubulin dimer. **b.** FSC curves for the Mal3-bound Sp_MT C1 reconstruction (dark green curve) and the symmetrized reconstruction (light green curve). **c,d.** Local resolution depiction using *blocres* [1] of the Mal3-bound Sp_MT symmetrized reconstruction. Views from **c**. the MT lumen and **d.** the side of a protofilament with the plus end to the right of the image and the minus end to the left.

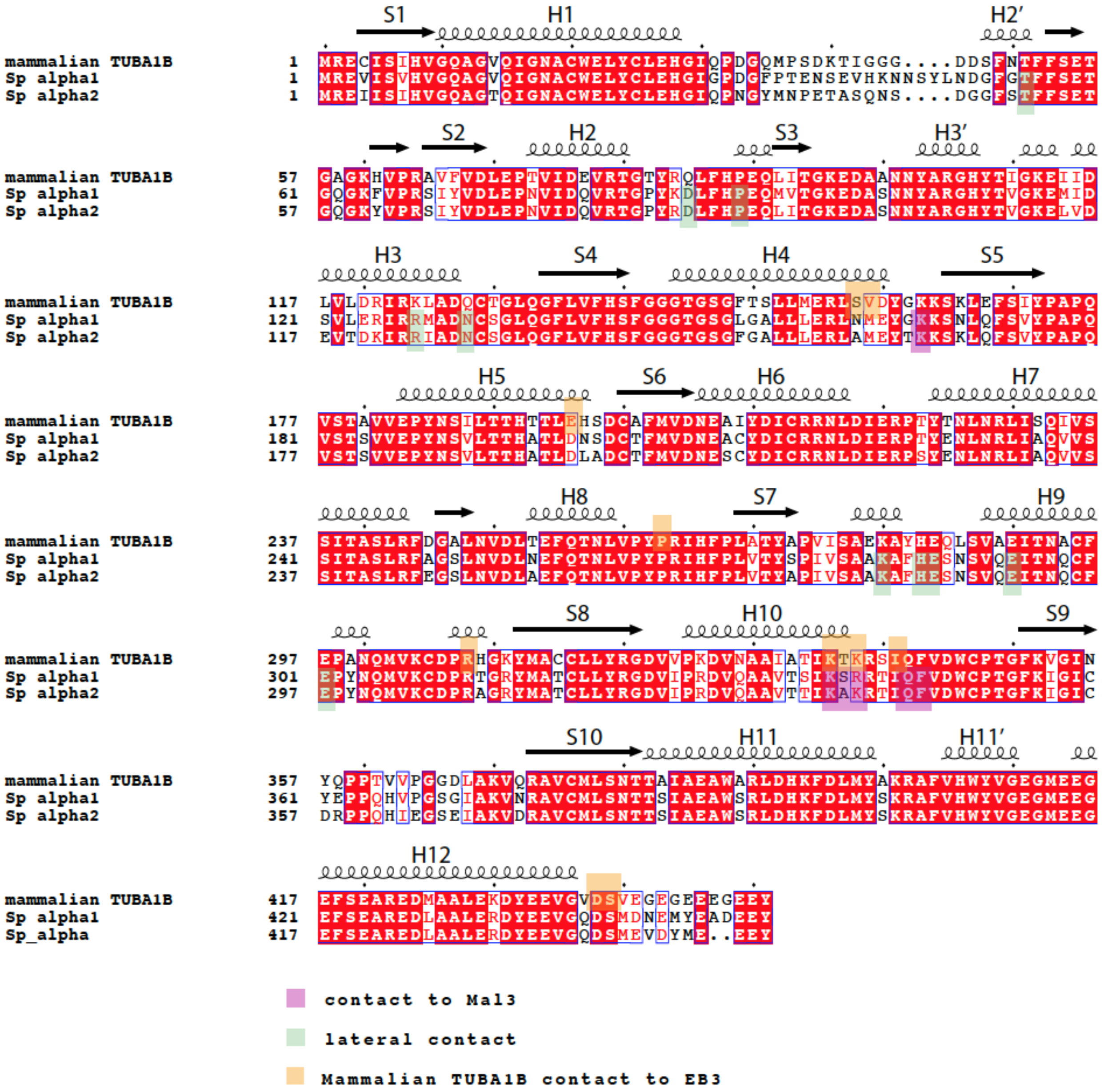


**Supplementary Figure 2. Sequence alignment of *S. pombe* α-tubulin 1 and 2 with mammalian TUBA1B.** Residues involved in lateral contacts (green) or contacting Mal3 (purple) in the Sp_tub MT structure, and residues of the mammalian (*S. scrofa)* tubulin contacting EB3 (orange) in 3JAR were calculated using PISA and are highlighted. Secondary structural elements in tubulin are depicted and labeled above the sequences.

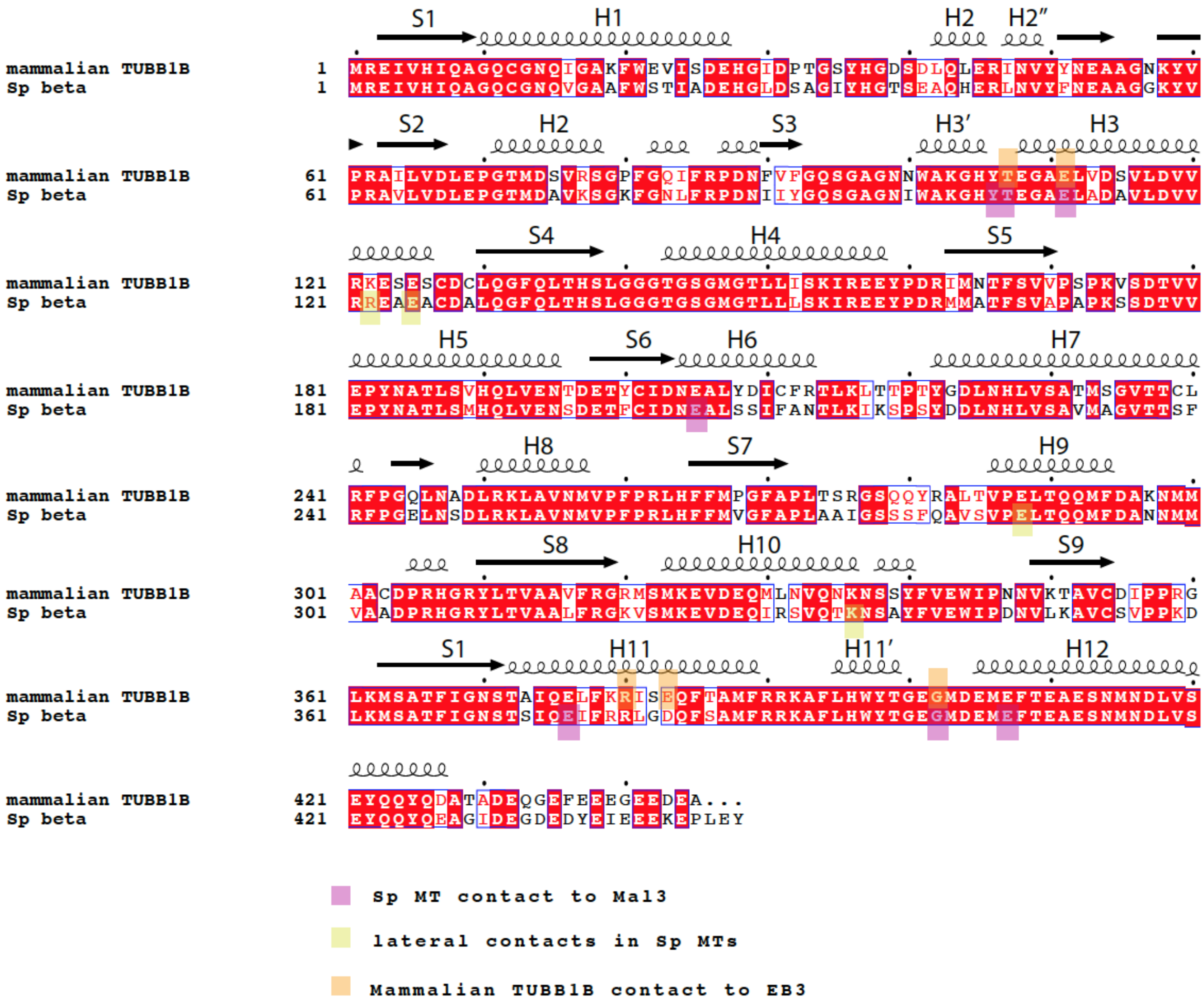


**Supplementary Figure 3. Sequence alignment of *S. pombe* β-tubulin-1 with mammalian TUBB1B.** Residues involved in lateral contacts (light green) or contacting Mal3 (purple) in the Sp_tub MT structure, and residues of the mammalian (*S. scrofa)* tubulin contacting EB3 (orange) in 3JAR were calculated using PISA and are highlighted. Secondary structural elements in tubulin are depicted and labeled above the sequences.

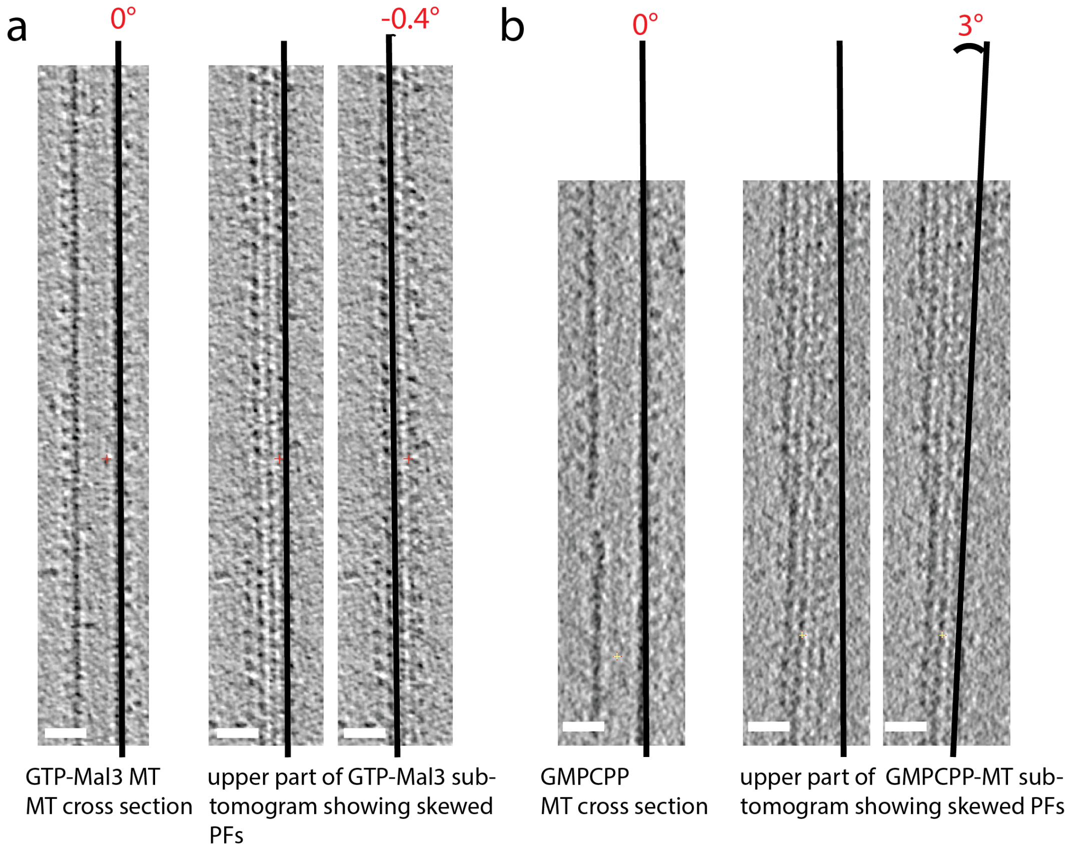


**Supplementary Figure 4. Visualization of PF skew in *S. pombe* MTs in slices through cryo-electron tomograms.** The straight MT walls in slices through the central section of cryo-ET reconstructions (left image) are compared manually, using the slicer function in IMOD, with the PF skew on the MT surface (right pair of images) in **a.** GTP-Mal3 MTs (+KMD as a marker of the lattice), where the skew is relatively small and **b.** GMPCPP MTs (+KMD as a marker of the lattice), where the PFs are more skewed. Scale bar = 20 nm.

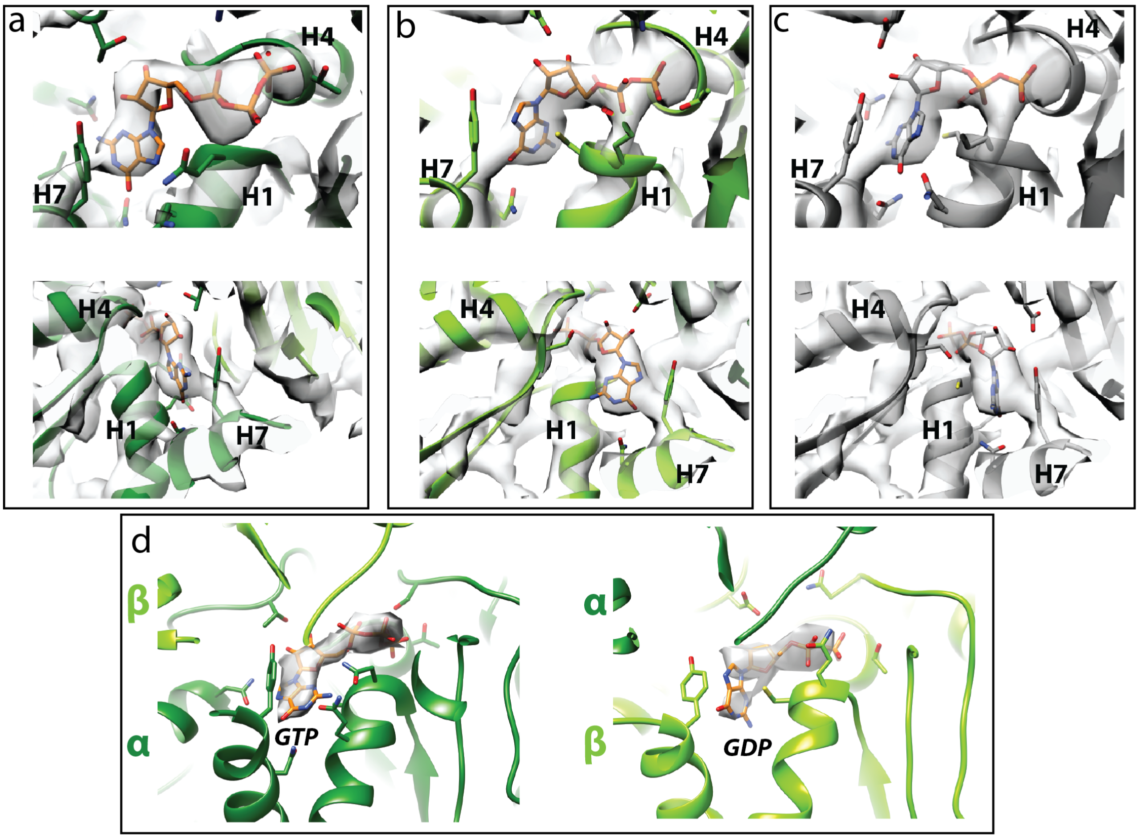


**Supplementary Fig. 5. The Sp_tub nucleotide binding sites.** Alternative views (top, bottom, in addition to Fig. 4c-e) of the nucleotide binding sites in the Mal3+Sp_tub reconstruction **a.** Mal3+Sp_tub α-tubulin N-site with Sp_tub molecular model docked (dark green). **b.** Mal3+Sp_tub β-tubulin E-site with Sp_tub molecular model docked (light green). **c.** Mal3+Sp_tub β-tubulin E-site with EB3+Mam_tub molecular model docked (grey) [+GTPγS]. **d.** Ribbon depiction of the atomic model of the Sp_tub MT N-site (left) and E-site (right) with density corresponding to the bound nucleotide shown in surface representation. This density is the calculated difference between our cryo-EM reconstruction and simulated 4.6 Å resolution density from the atomic models, calculated using Chimera [2]. This supports the conclusion that the E-site nucleotide in Sp_tub MTs is GDP and, thus, that GTP hydrolysis has occurred in these MTs despite the absence of lattice compaction.

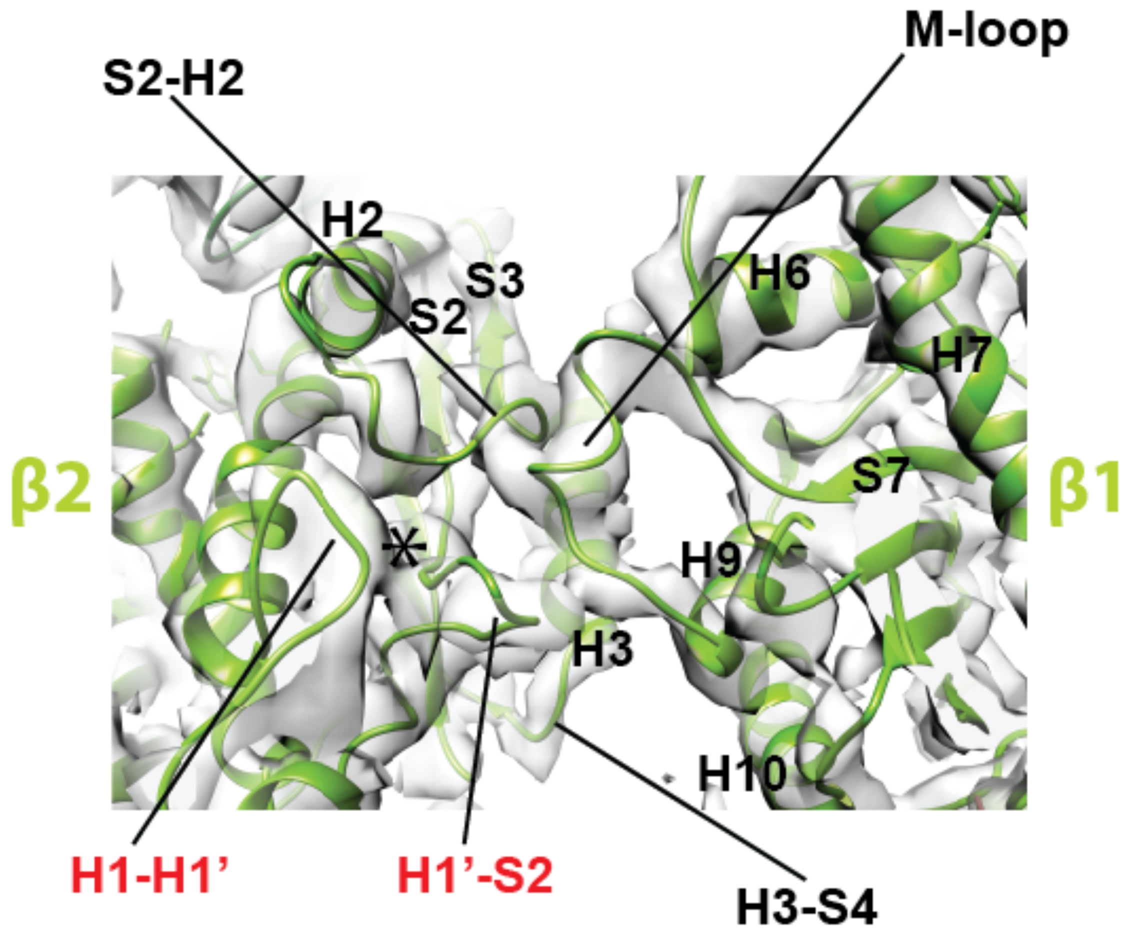


**Supplementary Figure 6. Connectivity in the N-terminal region of β-tubulin supports lateral contact stabilization.** Density (indicated with red asterisk) connects the H1-H1' loop – a site of *S. pombe*-specific sequence substitutions (Supplementary Fig. 3) - with the H1'-S2 lateral loop in β-tubulin.

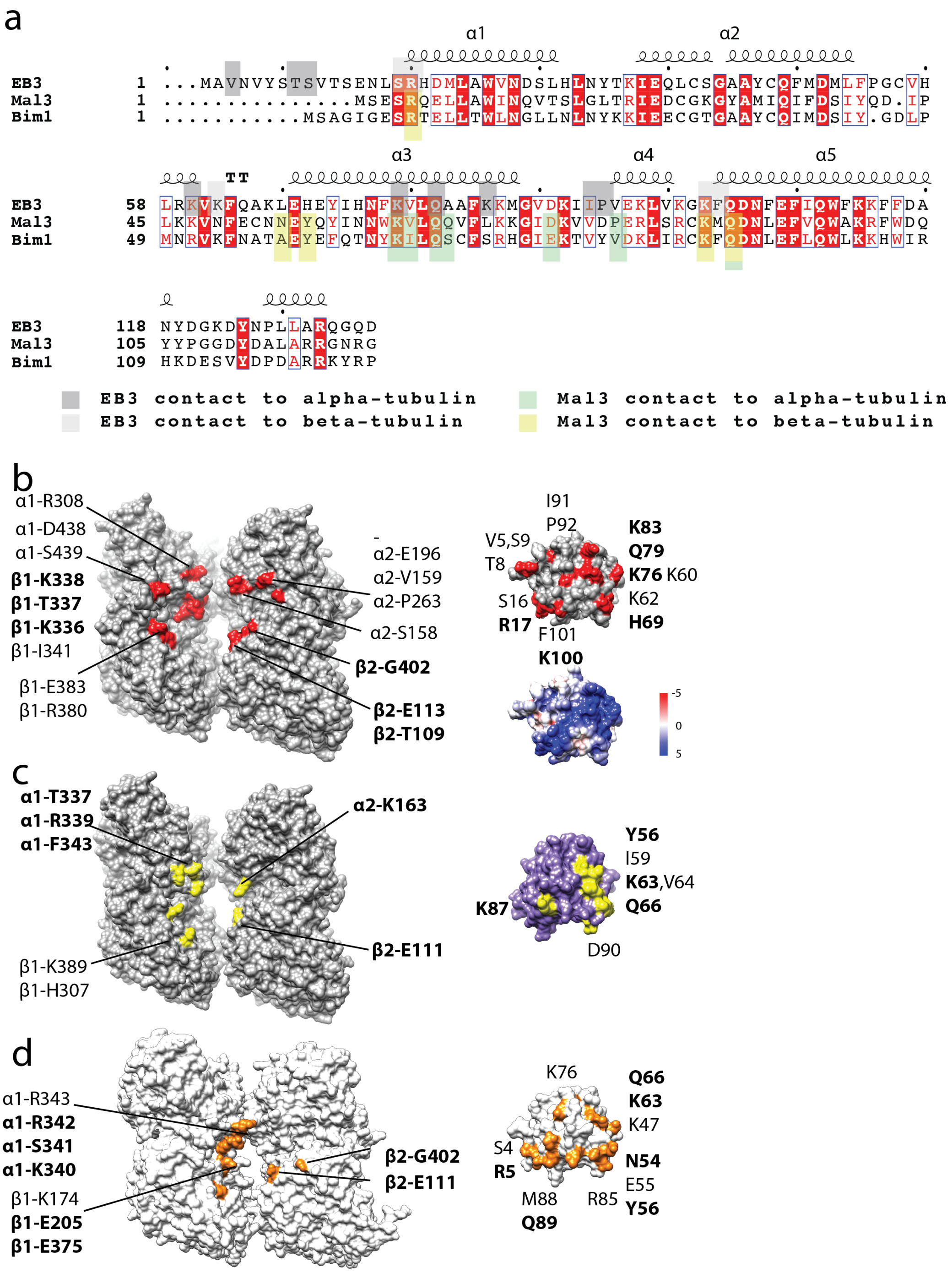


**Supplementary Figure 7. Comparison of EB MT binding patterns. a.** Sequence alignment of CH domains from EB3, Mal3 and Bim1. Mal3 residues contacting Sp_α-tubulin (dark green) and Sp_β-tubulin (light green) and residues of EB3 contacting Mam_α-tubulin (dark gray) and Mam_β-tubulin (light gray) are highlighted. **b,c,d** Footprints of EB3 (**b**) and Mal3 (**c,d**) interactions with MTs. The MT binding surface is shown on the left and the MT binding surface of EB3/Mal3 is shown on the right. Residues that are either identical or similar in b,c,d to the ones observed in our structure are printed in bold. **b.** Interactions of EB3 with mammalian MTs (PDB: 3JAR). Interacting residues are highlighted in red. In addition the surface charges of EB3 are shown. Indicating that most interaction residues are positively charged. **c.** Interactions of Mal3 with mammalian MTs (PDB: 4ABO). Interacting residues are highlighted in yellow. **d.** Predicted interactions of Mal3 to a compacted Sp_tub MT (homology model to 3JAR). Interacting residues are highlighted in orange.

| ***S. pombe* tubulin polymerisation conditions [no KMD added]** | | **PF number** | **moiré repeat (nm)** (±~2 nm) | **MT number (%)** |
|---|---|---|---|---|
| **GTP** | **-Mal3 *(n=25)*** *(same data set as in Table 1)* | 12 | 140-224 | 2 (8%) |
| | | 13 | 210- >1000 | 21 (84%) |
| | | 14 | 505-624 | 2 (8%) |
| | **+Mal3 (n=17)** | 13 | 520 ->1000 | 14 (82%) |
| | | 14 | 520 ->1000 | 3 (18%) |
| **GMPCPP** | **-Mal3 *(n=81)*** | Not measured | 90-170 | Not measured |
| | **+Mal3** Not measured | - | - | - |

**Supplementary Table 1. Effect of polymerization conditions on Sp_tub MT architecture.** MT architecture parameters were determined by analysis of 2D cryo-EM projection images (GMPCPP) or cryo-ET data in the absence of added KMD to confirm that KMD addition had no effect on the underlying MT architecture.

| | |
|---|---|
| RMSD (bonds) | 0.01 |
| RMSD (angles) | 1.13 |
| All atoms clashscore | 12.63 |
| Ramachandran outliers | 0.03% |
| Ramachandran allowed | 10.22% |
| Ramachandran favoured | 89.75% |
| Rotamer outliers | 0.66% |

**Supplementary Table 2. Refinement statistics and model geometry for the Mal3-Sp_tub model.**

**Supplementary References**

1. Cardone, G., Heymann, J.B. & Steven, A.C. One number does not fit all: mapping local variations in resolution in cryo-EM reconstructions. *J Struct Biol* **184**, 226-36 (2013).

2. Pettersen, E.F. et al. UCSF Chimera--a visualization system for exploratory research and analysis. *J Comput Chem* **25**, 1605-12 (2004).
