# Supplementary material for: Nucleotide– and Mal3-dependent changes in fission yeast microtubules suggest a structural plasticity view of dynamics
Source: Nat Commun. 2017 Dec 13;8:2110. doi: 10.1038/s41467-017-02241-5 (PMC5727398; doi:10.1038/s41467-017-02241-5)
Supplement: Supplementary file 4 — Description of Additional Supplementary Files [file 41467_2017_2241_MOESM4_ESM.pdf]

Descriptions of Additional Supplementary Files

File Name: Supplementary Movie 1
Description: Near-atomic resolution reconstruction of Mal3-bound Sp_MT with atomic model docked within the cryo-EM density. A 2PF section of the Mal3-Sp_tub MT reconstruction (white volume) shows the Mal3 CH domain (purple)bound at the corner of four tubulin dimers, with contacts made to two α-tubulins (dark green) and two β-tubulins (light green). The structure rotates to show the lumenal surface of the MT, with the change in transparency of the cryo-EM density emphasising the difference between α- and β-tubulin in their S9-S10 loops, a quality control for correct alignment of MTs in single particle reconstructions.
